# Ruminant versus non-ruminant manure composting: carbon-to-nitrogen ratio and associated physicochemical traits impact divergent pollutant emissions

**DOI:** 10.3389/fmicb.2026.1890442

**Published:** 2026-09-25

**Authors:** Conghui Zhang, Zhengkang Chen, Yu Cai, Jing Yuan, Guoying Wang, Guoxue Li

**Affiliations:** 1 State Key Laboratory of Environmental Criteria and Risk Assessment, Chinese Research Academy of Environmental Sciences, Beijing, China; 2 Research Center for Eco-Environmental Sciences, Chinese Academy of Sciences, Beijing, China; 3 Beijing Key Laboratory of Farmland Soil Pollution Prevention and Remediation, College of Resources and Environmental Science, China Agricultural University, Beijing, China

**Keywords:** aerobic composting, bacterial community, carbon-to-nitrogen ratio, gaseous emissions, ruminant manure

## Abstract

Composting can convert livestock manure into valuable fertilizer, but the relationships between manure type, which essentially reflects differences in carbon-to-nitrogen ratio (C/N) and other associated physical properties, and pollutant gas emissions remain poorly quantified. We monitored temperature, pH, carbon, nitrogen, sulphur contents, bacterial succession, and emissions of methane (CH_4_), nitrous oxide (N_2_O), ammonia (NH_3_) and hydrogen sulfide (H_2_S) during aerobic composting of non-ruminant manure (chicken and pig) and ruminant manure (sheep and cattle). Sheep and cattle manure (characterized by higher C/N) reached maturity earlier and emitted 30–93% less greenhouse gases and odor than chicken and pig manure (characterized by lower C/N), although cattle manure produced slightly more CH_4_. High emissions from chicken and pig manure coincided with dominance of *Bacillus* and *Defluviitalea*, which declined as readily available carbon was depleted. In contrast, sheep and cattle manure was associated with *Actinomadura* and *Corynebacterium*, genera that correlated with the immobilization of nitrogen and sulphur into stable forms during the degradation of recalcitrant carbon. Ammonium concentration was strongly correlated with the overall emission rate, yet the microbial taxa associated with the process shifted predictably with animal diet. The observed associations provide a basis for future hypothesis-driven experiments. The findings provide a microbiome-based guide for controlling and minimizing atmospheric pollution during composting.

## 1 Introduction

China produces as much as 3.8 billion tons of livestock manure annually, but its comprehensive utilization rate is less than 60% (Zhang et al., 2023). Aerobic composting, as a common method of organic waste utilization, transforms organic waste into stable organic fertilizer (Wang et al., 2024). However, the generation of undesirable gases, mainly odor-causing ammonia (NH_3_) and hydrogen sulfide (H_2_S) and greenhouse gas of methane (CH_4_) and nitrous oxide (N_2_O), has primarily inhibited the large-scale application of composting (Deng et al., 2026). According to statistics, NH_3_ and N_2_O emissions from organic mineralization accounted for 16–74% of total nitrogen losses (Yin et al., 2021). CH_4_ and N_2_O release can cause 1–9% of nutrient loss, corresponding to greenhouse gas emissions of 180–250 g CO_2_-eq kg·VS^−1^ (Zhang, L., et al., 2025). H_2_S accounts for 39.0–43.0% of the total volatile sulphur compounds released during composting (Yuan et al., 2019). The emission of these pollutant gases not only reduces composting efficiency but also has significant impacts on global climate change and air quality.

Previous studies have explored the reduction effect of greenhouse gas and odor emissions under different conditions. Increasing temperature, enhancing aeration, improving carbon-to-nitrogen ratio (C/N), and adding additives can significantly reduce CH_4_ and N_2_O emissions by 25–50% during composting (Chung et al., 2021). Similarly, increasing aeration frequency, controlling composting temperature, and adjusting the C/N can significantly reduce NH_3_ and H_2_S emissions by 40–45% (Li, Y., et al., 2022). However, the type of livestock manure is a critical factor influencing pollutant gas emissions (Li, L., et al., 2023). Most studies have focused on the impact of reduction methods on gas emissions during composting based on one or two types of livestock manure (Chen et al., 2024), and research on the emission and reduction mechanisms of greenhouse gases and odors during composting of all major types of livestock manure in China remains lacking.

There are four major types of livestock manure in China, including chicken, pig, sheep and cattle manure, which differ in organic composition and C/N (Wang et al., 2015). Ruminants manure (cattle and sheep) contains abundant lignin, cellulose and hemicellulose, resulting in a fibrous, less viscous texture and higher C/N (Zhang et al., 2026). In contrast, manure from non-ruminants livestock (e.g., chicken and pig) is more granular and has a higher nitrogen content, leading to rapid decomposition and substantial nitrogen emissions when not balanced with sufficient carbon, as reflected by its relatively low C/N (Cao et al., 2021). Thus, composting with non-ruminants manure achieves lower and more variable reduction of greenhouse gas and odor emissions (15–60%), whereas ruminants manure can achieve up to 85.8% reduction (Duan et al., 2024). However, the specific mechanism of these emissions and their relationships with microorganisms and environmental factors remain unclear.

Microorganisms play a crucial role in greenhouse gas and odor emissions, and microbial activity is influenced by environmental factors during composting (Zhou et al., 2025). While ammonia-oxidizing bacteria can lower NH_3_ emissions, the N_2_O-mitigating effect of denitrifiers is pathway-dependent, only complete denitrifiers (possessing N_2_O reductase) reduce N_2_O to N_2_ (Lin et al., 2023; Zhou et al., 2022). Methanotrophic bacteria effectively reduce CH_4_ emissions by oxidizing methane, a temperature-sensitive process in environment (Jeffrey et al., 2021; Jiang et al., 2025). In addition, sulfate-reducing bacteria drive H_2_S flux under coupled hypoxia-acid-heat-oxidative stress during composting (Liu et al., 2025). Thus, microorganisms and environmental factors operate synergistically to attenuate greenhouse gas and odor emissions during composting.

This study was designed to (i) quantify and compare the temporal profiles of CH_4_, N_2_O, NH_3_ and H_2_S emitted during composting of non-ruminant (chicken, pig) and ruminant (sheep and cattle) manure, (ii) identify the bacterial taxa whose dynamics coincide with each gas pulse, and (iii) elucidate the combined influence of composting properties (C/N, ammonium, pH, electrical conductivity, temperature, maturity) and these keystone microbes on pollutant release. This study unveils a microbially mediated, operationally relevant pathway for mitigating greenhouse gas and odor emissions across four mainstream livestock manure composting.

## 2 Materials and methods

### 2.1 Raw materials collection and characteristics

The main feedstocks of composting were chicken manure, pig manure, sheep manure and cattle manure. Garden waste was used as a bulking agent. Chicken manure and pig manure were obtained from Chinese Academy of Agricultural Sciences (Beijing, China). Sheep manure was obtained from Aoxin Sheep Farm (Beijing, China), and cattle manure was obtained from Langfang Cattle farm (Hebei, China). All animals were raised on commercial farms, and manure was collected without bedding material or urine. Garden waste was collected from Meishang Ecological Landscaping Company (Jiangsu, China), crushed to 2–3 cm and air-dried before composting. The basic physicochemical properties of the raw materials are shown in Table 1.

**Table 1:** Physicochemical properties of raw materials.

Characteristic	Chicken manure	Pig manure	Sheep manure	Cattle manure	Garden waste
Moisture content (%)^a^	74.38 ± 0.16	68.77 ± 2.22	54.86 ± 3.18	71.07 ± 1.30	15.53 ± 2.33
pH^a^	6.79 ± 0.04	6.86 ± 0.04	7.17 ± 0.03	7.28 ± 0.09	7.32 ± 0.03
EC (mS·cm^−1^)^a^	7.18 ± 0.13	3.21 ± 0.06	4.01 ± 0.01	3.17 ± 0.06	0.31 ± 0.02
C/N^b^	10.20 ± 0.21	13.59 ± 0.66	20.37 ± 1.19	22.17 ± 0.24	64.36 ± 1.26
Organic matter (%)^b^	82.63 ± 0.35	83.67 ± 0.19	86.01 ± 0.04	81.61 ± 0.03	83.20 ± 0.84
NH_4_^+^ (g·kg^−1^)^b^	12.01 ± 0.75	11.68 ± 0.11	6.56 ± 0.61	1.65 ± 0.11	0.84 ± 0.01

^a^Wet-weight basis; ^b^Dry-weight basis. EC: electrical conductivity, C/N: carbon-to-nitrogen ratio.

### 2.2 Experimental design

To compare the four manure types, we prepared composting mixtures by combining each manure with garden waste in a 3:7 ratio (wet-weight basis). Four treatments were established and denoted as CM, PM, SM and CoM for chicken manure, pig manure, sheep manure and cattle manure composting, respectively. The initial moisture content was adjusted to 65% with deionized water, and continuous aeration was provided at 0.27 L·kg^−1^·min^−1^with an air pump. The initial mass of each wet mixture was 30 kg, and the composting duration was 49 days (Wang et al., 2024).

Composting was conducted in a 60-L cylinder (60 cm in height and 36 cm inf inner diameter), which had two layers of stainless steel to minimize heat loss. Each composter had a stainless-steel lid with two holes for gas release and temperature sensor insertion. A stainless-steel grid with uniform pores (3 mm diameter) was placed at the bottom of the composter to ensure homogenous air supply. Two holes at the bottom allowed for aeration and leachate drainage. Details of the composting system have been described elsewhere (Wang et al., 2024).

The composting mixture was manually turned and sampled on day 0, 7, 14, 28, 39, and 49. Turning involved approximately 10 shovel-mixing repetitions every 7 days to ensure uniform blending. After each turning, composite samples were collected using the five-point method from three layers (upper, middle, lower), with 10 subsamples per layer, which were then pooled into one representative sample per time point. A total of 300 g of samples was collected and divided into three parts. One part was air-dried and sieved to <0.2 mm for determination of organic matter and elemental contents. The second part was stored at 4 °C for the physicochemical parameter measurements. The remaining part was stored at −20 °C for bacterial community analysis.

The gas released from each composter was collected daily for measurement of CH_4_, N_2_O, NH_3_ and H_2_S. CH_4_ and N_2_O were sampled using 1-L aluminum foil gas bags, each filled within 15–20 s. NH_3_ was trapped in 2 M boric acid solution with methyl red–bromocresol green indicator until the solution turned from blue to red; trapping time ranged from <10 s in the early stage to ≥3 min in the later stage. H_2_S was measured directly on-site using a portable gas analyzer without bag collection.

### 2.3 Analytical methods

#### 2.3.1 Physiochemical properties

Temperature was recorded at 9:00 a.m. every day using automatic digital thermometers (WD-6210, Beijing Hong Yuan Peng Ao Co., Ltd., China). Fresh compost samples were extracted with deionized water at a 1:10 (w/v) ratio, and the resulting suspensions were shaken, filtered and used for pH, electrical conductivity (EC) and germination index (GI) measurements. The pH and EC were determined using a pH/EC meter (pHS–3C, Shanghai Precision & Scientific Instrument Co., Ltd., China), and GI was calculated based on the germination rate and radical length of radish seeds. Potassium chloride was used instead of deionized water to prepare extracts for nitrate (NO_3_^−^) and ammonium (NH_4_^+^) determination using a segmented flow analyzer (Seal XY-2 Sampler, Australia). Air dried samples were used for total carbon (TC), total nitrogen (TN) and total sulphur (TS) determination using an elemental analyzer (Elementar Analysensysteme, Hanau, Germany).

#### 2.3.2 Pollutant gas emission measurements

CH_4_ and N_2_O concentrations were determined using a gas chromatograph (Trace 1310, thermo Fisher Scientific, USA) equipped with a flame ionization detector (FID, for CH_4_) and an electron capture detector (ECD, for N_2_O), with a Porapak Q column (2 m × 3 mm, 80/100 mesh) at 60 °C, and N_2_ or Ar/CH_4_ (95/5) as carrier gas (30 mL·min^−1^). H_2_S concentrations were measured directly *in situ* using a portable biogas analyzer (Biogas 5000, Geotech, UK) equipped with an electrochemical sensor (range: 0–5,000 ppm; T90 response time <30 s). Cumulative gas emissions were calculated using the following formula Cumulative gas emissions were calculated using the following formula (1):

F(g·d−1)=101.325×1000×Q×24×(b−b0)×10^−6×M8.314×(273+T0) (1)

Where *Q* is the aeration rate (m^3^·h^−1^); *b* is the measured gas concentration gas (ppm), *M* is the molar mass of gas (16.04 g·mol^−1^ for CH_4_, 44.01 g·mol^−1^ for N_2_O, 34.06 g·mol^−1^ for H_2_S), and *T*_0_ is the ambient temperature (°C).

NH_3_ was absorbed in 2% boric acid and quantified by titration with 0.01 mol·L^−1^ dilute sulfuric acid. The NH_3_ emission rate was calculated using the following formula The NH_3_ emission rate was calculated using the following formula (2):

$$F\;(g\cdot d^{-1})=\frac{C\times (V-V_{0})\times M}{N}\times \frac{60}{t}\times 24$$ (2)

Where *C* is the concentration of the standard sulfuric acid solution (mol·L^−1^), *V* is the volume of sulfuric acid consumed (mL), *V*_0_ is the volume consumed in the blank test (mL), *M* is the molar mass of NH_3_ (17.03 g·mol^−1^), *t* is the absorption time (h), and *N* is the dry weight of the compost (kg).

#### 2.3.3 High-throughput sequencing

Genomic DNA of all samples was extracted according to the instructions of the FastDNA SPIN Kit for Feces (MP Biomedicals, Santa Ana, USA). DNA concentration and purity were assessed using a spectrophotometer (NanoDrop ND-1000, Thermo Fisher Scientific, USA). Primers 341F/798R (TACGGRAGGCAGCAG and AGGGTATCTAATCCT) were selected for polymerase chain reaction to amplify the V3–V4 conserved regions. Sequencing was performed on the Illumina MiSeq PE250 platform, and sequencing service was provided by Oebiotechnology Co. Ltd. (Shanghai, China).

### 2.4 Statistical analyses

All parameters were measured in triplicate from samples collected from each composting reactor, and the results are expressed as means ± standard deviations of these technical replicates. Statistical significance was assessed using one-way ANOVA with Tukey’s test for multi-group comparisons and Spearman’s correlation for pairwise associations, with *p* < 0.05 considered statistically significant. Bubble plots were generated in Origin 2026 to visualize the relative abundances of the top 30 genera (defined as dominant genera) across treatments. Co-occurrence networks, delineating associations between microbial genera (defined as key genera, relative abundance >0.1%) and gas fluxes, were constructed based on Spearman correlation coefficients (|*ρ*| > 0.6, *p* < 0.05) using R version 4.3.0, with network visualization performed in Gephi 0.9.2. A partial least-squares path model (PLS-PM) was constructed using SmartPLS 4.0 to quantify the causal cascade linking environmental drivers and microbial biomass (defined as keystone genera) to gaseous emissions. The model comprised three latent constructs, each with multiple manifest indicators. Model fit was acceptable (*SRMR* < 0.05), blindfolding confirmed predictive relevance (*R*^2^ > 0.5), and all variance inflation factors (*VIF*) were below 3.0. Latent variables were constructed following established guidelines, including a minimum of three manifest variables per latent construct and a factor loading threshold of >0.5.

## 3 Results and discussion

### 3.1 Physicochemical parameters

Composting proceeded through three thermally distinct phases (Figure 1a), an initial mesophilic-to-thermophilic phase (0–14 d), a sustained cooling phase (15–35 d), and a subsequent maturation sequence (36–49 d). Maximum temperatures recorded for CM, PM, SM and CoM were 72.1 °C, 74.0 °C, 74.7 °C and 68.1 °C, respectively. Although CM and PM attained comparable thermophilic peaks, SM and CoM retained significantly higher temperatures during the cooling (14–28 d) and maturation (28–49 d) stages (*p* < 0.05). This thermal persistence is attributed to the prolonged exothermic decomposition of cellulose- and hemicellulose-rich constituents that dominate ruminants feedstocks (Wan et al., 2021). Notably, CoM exhibited a secondary temperature rebound during the final 7 days, implying delayed microbial attack on the recalcitrant lignin matrix and associated release of metabolic heat.

**Figure 1:**
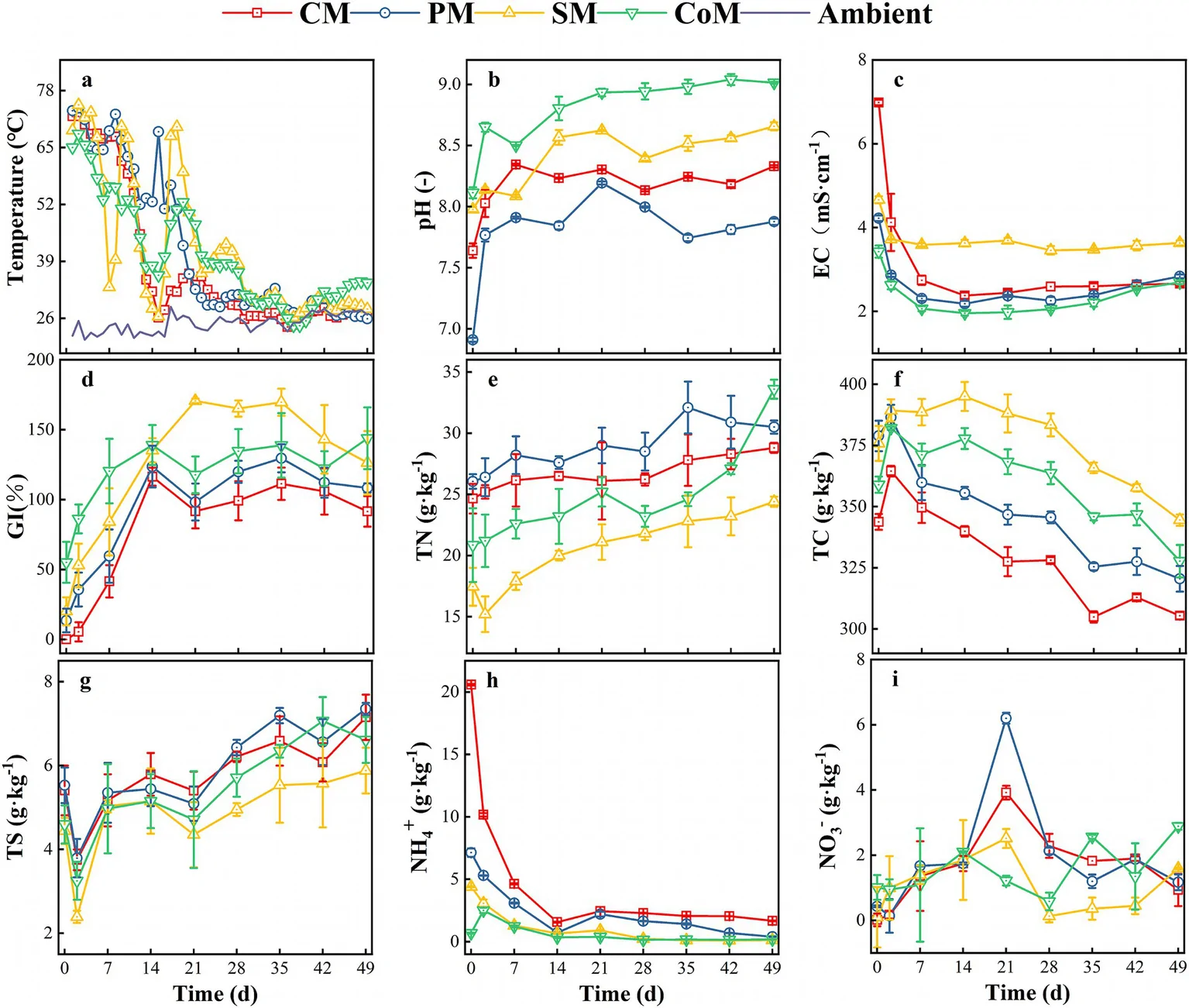
Changes in physicochemical properties during composting. **(a)** temperature, **(b)** pH, **(c)** EC, **(d)** GI, **(e)** TN, **(f)** TC, **(g)** TS, **(h)** NH_4_^+^ and **(i)** NO_3_^−^ content. EC: electrical conductivity, GI: germination index, TN: total nitrogen, TC: total carbon, TS: total sulphur. CM: chicken manure, PM: pig manure, SM: sheep manure, CoM: cattle manure composting.

pH rose steadily in all piles (Figure 1b) as organic acids were decarboxylated, NH_3_ volatilized and humification consumed protons (Pang et al., 2023). Alkalization followed the order CoM > SM > CM > PM (*p* < 0.05), lower acidogenic organic and higher free air space accelerated NH_4_^+^-to-NH_3_ conversion in ruminant manure piles. PM alone showed a transient pH dip on days 21–35, when turning-induced mineralization accumulated phenolics and amino acids, coinciding with a minor heat rebound (Figure 1a) and potential N_2_O bursts via denitrification (Umar et al., 2025).

EC in SM consistently exceeded that in the other matrices (Figure 1c) because sheep rations are routinely supplemented with salt, which is predominantly composed of Na^−^ and Cl^−^. The initial EC of CM (6.98 mS·cm^−1^) was likewise elevated, reflecting the high protein–derived nitrogen in chicken manure that rapidly mineralizes to NH_4_^+^ and NO_3_^−^ (Manu et al., 2021). During the maturation phase, EC in PM and CoM rose marginally as continued organic matter mineralization released low-molecular-weight soluble salts (Wan et al., 2020). Nevertheless, final EC values in all composts remained <4 mS·cm^−1^, complying with the phytotoxicity threshold for safe agronomic use (Yan et al., 2023).

GI followed a rapid ascent with minor oscillations in all piles (Figure 1d). The initial 14-day surge reflects the parallel exhaustion of phytotoxic short-chain organic acids and evaporation of NH_3_, two principal germination inhibitors (Wang et al., 2022). Subsequent fluctuations on day 21 mirror transient accumulations of residual volatile fatty acids and pulse releases of NH_4_^+^ during incomplete organics mineralization. Peak GI (116.38–171.62%) was recorded on day 35, coinciding with the maximum humification rate. During the maturation phase, GI marginally declined in CM, PM and SM, especially in SM, which might be associated with elevated soluble nitrogen (including NO_3_^−^) and low-molecular-weight organic acids (Wang et al., 2022; Kong et al., 2022). Conversely, CoM exhibited a significant 7-day GI upshift (*p* < 0.05), reflecting superior humification and advanced phytotoxic detoxification (Kong et al., 2023). Nevertheless, terminal GI exceeded the 70% phytotoxicity threshold in every treatment, confirming compost maturity (Ji et al., 2023).

TN and TS concentrations increased across all treatments while TC declined (Figures 1e–g). CM and PM exhibited higher TN and TS but lower TC than SM and CoM, reflecting greater protein relative to lignocellulose content (Li, L., et al., 2023). Absolute contents of TN, TC and TS decreased throughout composting (Table 2), with mass-loss rates ordered TC > TN > TS, losses from CM and PM exceeded those from SM and CoM. These patterns highlight divergent organic matter mineralization between non-ruminant and ruminant manures. Nitrogen loss occurred mainly as NH_3_-N emissions, which represented 11.5–36.5% of initial TN, whereas less than 1% of TC and TS was released as CH_4_ and H_2_S, respectively.

**Table 2:** Elemental balance during composting.

Treatment		CM	PM	SM	CoM
Dry matter	Initial (kg)	11.078	12.419	15.795	13.186
	End (kg)	4.644	4.921	6.420	5.882
	Loss ratio (%)^a^	58.080	60.372	59.355	55.390
TN	Initial (kg)	0.273	0.324	0.276	0.275
	End (kg)	0.134	0.150	0.157	0.198
	NH_3_-N (%)^b^	36.564	30.774	19.996	11.508
	N_2_O-N (%)^c^	0.446	1.156	0.166	0.095
	Loss ratio (%)^a^	51.022	53.602	43.167	28.111
TC	Initial (kg)	3.808	4.706	5.936	4.733
	End (kg)	1.418	1.577	2.212	1.928
	CH_4_-C (%)^d^	0.006	0.005	0.003	0.006
	Loss ratio (%)^a^	62.756	66.479	62.741	59.274
TS	Initial (kg)	0.060	0.069	0.070	0.061
	End (kg)	0.033	0.036	0.038	0.039
	H_2_S-S (%)^e^	0.351	0.709	0.210	0.182
	Loss ratio (%)^a^	44.546	47.329	46.173	35.828

^a^ $\text{loss ratio}\;(\%)=\frac{\text{Initial absolute content}-\mathrm{End}\;\text{absolute content}}{\text{Initial absolute content}}\times 100$ , ^b^the ratio of N loss in the form of NH_3_ compared with the initial TN, ^c^the ratio of N loss in the form of N_2_O compared with the initial TN, ^d^the ratio of C loss in the form of CH_4_ compared with the initial TC, ^e^the ratio of S loss in the form of H_2_S compared with the initial TS. TN: total nitrogen, TC: total carbon, TS: total sulphur. CM: chicken manure, PM: pig manure, SM: sheep manure, CoM: cattle manure composting.

Inorganic nitrogen was dominated by NH_4_^+^ and NO_3_^−^, whose trajectories were mirror-imaged throughout composting (Figures 1h,i). Thermophilic NH_3_ volatilization coupled with nitrifier-driven oxidation steadily lowered NH_4_^+^ while enriching NO_3_^−^ (Hussain et al., 2023). CM and PM, richer in total nitrogen (Table 1), released more NH_4_^+^ initially, whereas SM and CoM showed faster NO_3_^−^ build-up at 28–49 days (Figure 1i), indicating rapid nitrification. The recalcitrant lignocellulosic scaffold of ruminant manures simultaneously immobilized mineral nitrogen and created micro-aerobic niches, partially attenuating NO_3_^−^ formation (Wang et al., 2023).

### 3.2 Gas emission patterns

A pronounced CH_4_ emission peak was observed across all treatments during the initial composting phase (Figure 2a), attributed to the presence of anaerobic microflora, particularly acidogens and methanogens (Essonanawe Edjabou and Scheutz, 2023). Subsequently, emissions increased gradually from day 2, reaching maxima on days 13–14. This transient rise coincided with a progressive decline in thermophilic conditions, which otherwise suppress methanogenic activity, and with the drop in core temperature. Notably, SM amendment attenuated cumulative CH_4_ emissions by 30–41% relative to other piles (Figure 2b). This mitigation reflects a sustained thermophilic phase, low moisture (Supplementary Figure S1a), and ellipsoidal particles of SM that together suppress CH_4_ generation by limiting anaerobic niches and enhancing oxygen diffusion (Liu, H., et al., 2024). Conversely, CoM, despite its comparable recalcitrant organic load (e.g., cellulose), exhibited higher CH_4_ yield, primarily because its lower bulk density and elevated moisture fostered localized anaerobic fermentation zones (Zhang, L., et al., 2023). Similarly, CM and PM, rich in readily hydrolysable organics (carbohydrates, proteins, lipids), promoted CH_4_ emission under the microaerobic conditions prevailing within their respective composting matrices (Dang et al., 2024).

**Figure 2:**
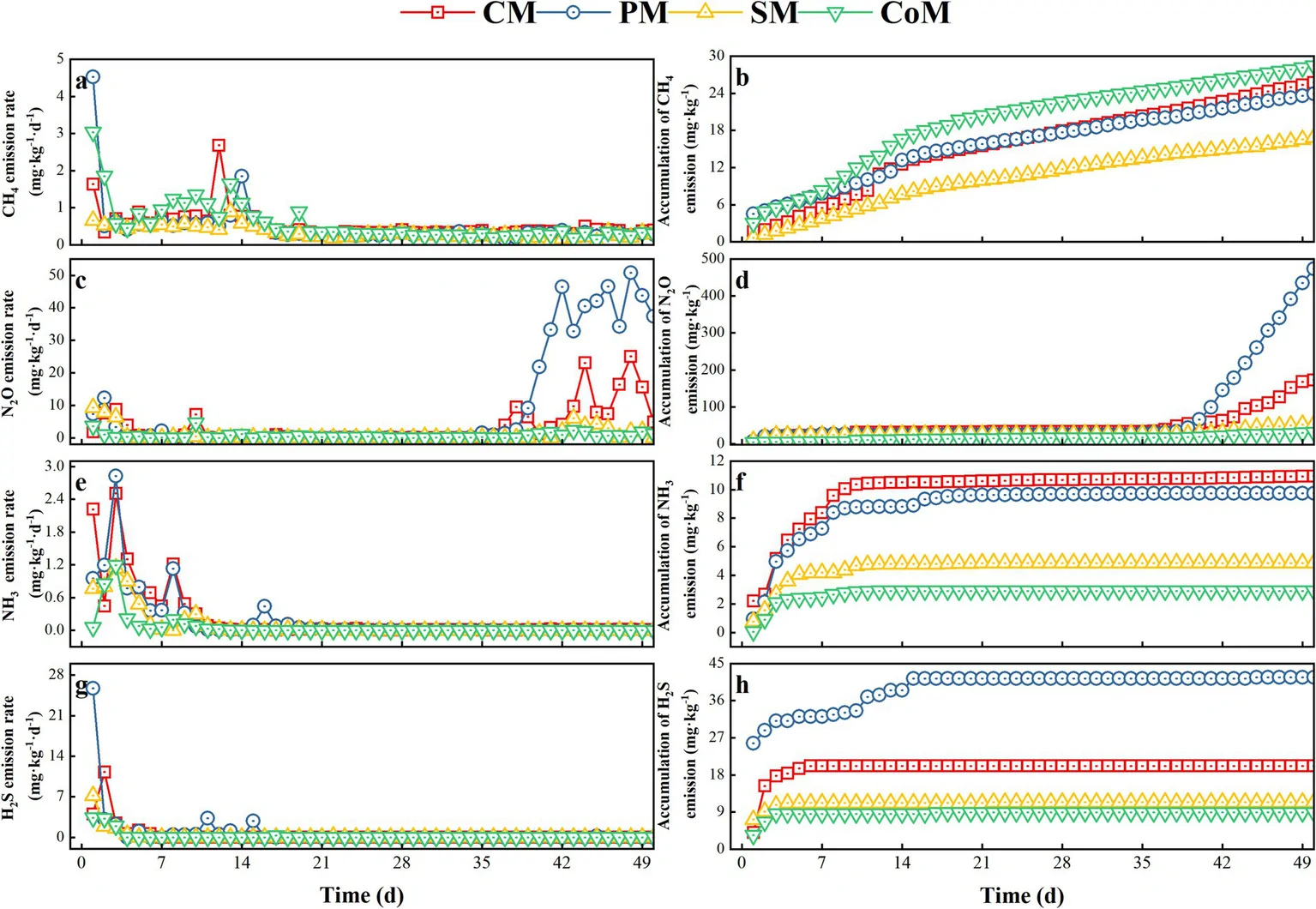
Greenhouse gases and odors emission during composting. **(a)** daily and **(b)** accumulative emission of CH_4_, **(c)** daily and **(d)** accumulative emission of N_2_O, **(e)** daily and **(f)** accumulative emission of NH_3_, **(g)** daily and **(h)** accumulative emission of H_2_S. CM: chicken manure, PM: pig manure, SM: sheep manure, CoM: cattle manure composting.

CM and PM exhibited pronounced N_2_O emission during the maturation phase (Figure 2c), mirroring the concomitant NO_3_^−^ accumulation (Figure 1i) that fuelled denitrification. Thermophilic temperatures transiently suppressed denitrifier activity; the subsequent temperature decline reactivated the pathway, triggering intensive N_2_O release. Consequently, cumulative N_2_O from SM and CoM was reduced by 70–93% compared with that from CM and PM (Figure 2d). The high C/N and low TN of SM and CoM (Table 1) curtail nitrifier and denitrifier metabolism, thereby limiting N_2_O yield (Han et al., 2024). Non-negligible N_2_O was also recorded at the outset across all treatments. The elevated initial NH_4_^+^-N (Table 1) was rapidly channeled into coupled nitrification and denitrification reactions. Micro-scale anoxic clusters, favored by low C/N, further amplified net N_2_O production (Stegenta-Dąbrowska et al., 2022).

NH_3_ emissions from all treatments were concentrated in the first 14 days (>80% of the total loss, Figure 2e). The rapid temperature rise during this phase accelerated the conversion of organic nitrogen to NH_3_ (Qi et al., 2022). SM and CoM, which contained the lowest initial NH_4_^+^ (Figure 2f), lost 50–73% lower NH_3_ than CM and PM. This difference is largely explained by C/N, the higher C/N in SM and CoM ties up mineral nirogen and slows ammonification (Li, D., et al., 2022). In addition, the larger fraction of slowly degradable carbon (e.g., cellulose) in SM and CoM diverted microbial activity toward carbon decomposition, further reducing NH_3_ formation (Li, L., et al., 2023). Although CM and PM had slightly lower pH, the values were still alkaline (>7.8), sufficient to neutralize acidic substrates without suppressing NH_3_ release. CM emitted 12% more NH_3_ than PM because chicken have a shorter gut and less complete digestion, leaving more undigested nitrogen in the manure (Bao et al., 2022). This extra nitrogen was quickly mineralized to NH_4_^+^ and then lost as NH_3_ (Zhu et al., 2024a).

H_2_S emissions were concentrated in the first 3 days across all piles (Figure 2g). PM released the most H_2_S, peaking at 25.7 mg·kg^−1^·d^−1^ on day 1 and accumulating to 41.7 mg·kg^−1^ by the end of the composting. CM ranked second, with a maximum of 11.3 mg·kg^−1^·d^−1^ on day 2 and a total of 20.2 mg·kg^−1^. In contrast, SM and CoM showed modest peaks of 7.2 and 3.4 mg·kg^−1^·d^−1^, respectively, and cumulative losses of only 11.4 and 8.9 mg·kg^−1^, 44–79% lower compared with CM and PM. The high CO_2_ levels measured in CM and PM (Supplementary Figure S1b) created local anaerobic zones that favored sulfate-reducing bacteria. In addition, the greater protein content of chicken and pig manure provided abundant organic sulphur, which was rapidly mineralized to H_2_S during the initial acidogenic phase (Chen et al., 2019). Sheep and cattle manure, with lower protein and higher recalcitrant carbon, supplied less readily available sulphur and maintained better aeration, thereby suppressing H_2_S formation (Li et al., 2020).

### 3.3 Succession of bacterial communities

Chao1 and Shannon indices were used to track bacterial richness and diversity (Figures 3a,b). CM and PM showed the highest Chao1 and the lowest Shannon, meaning more species but lower evenness. This pattern matches the report by Kong et al. (2022) that non-ruminant gut microbiota is richer yet less diverse than that of ruminants. Chicken and pig manure contain larger pools of soluble carbon and nitrogen, including proteins, saccharides and amino acids, than ruminant manures, and these labile substrates promote rapid growth of a few dominant taxa (Wang et al., 2024). Richness and diversity increased gradually from day 28 to 49 without significant differences (*p* > 0.05). Both indices declined slightly during the thermophilic phase, although the decrease was not significant in PM and CoM, because high temperature suppressed heat-sensitive bacteria.

**Figure 3:**
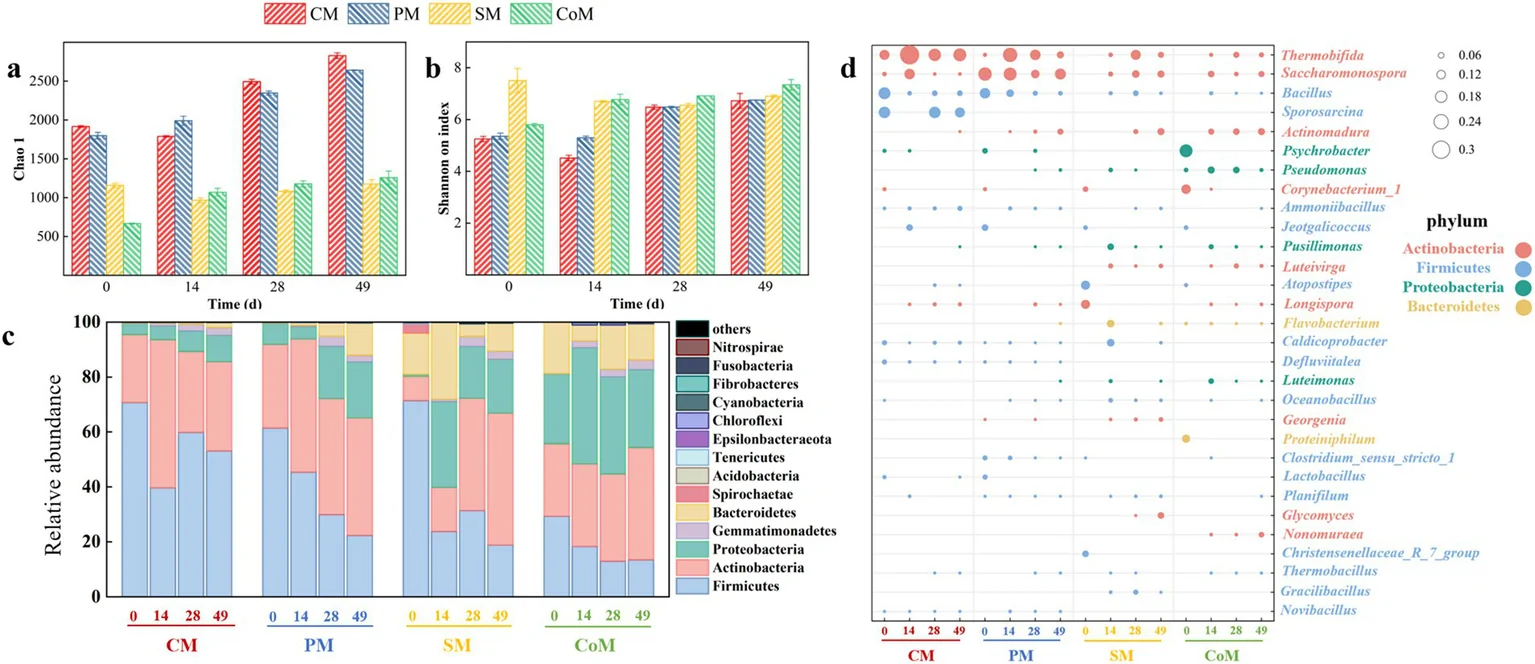
Bacterial communities during composting. Alpha diversity of **(a)** Chao1 and **(b)** Shannon indices, relative abundance at **(c)** phylum and **(d)** genus level. CM: chicken manure, PM: pig manure, SM: sheep manure, CoM: cattle manure composting.

The dominant bacterial phyla remained consistent across all treatments, comprising Firmicutes, Actinobacteria, Proteobacteria, and Bacteroidetes, although their relative abundances exhibited significant treatment-dependent variations (Figure 3c). Microbial community succession patterns were strongly associated with manure origin, clustering into two distinct groups based on animal feeding strategies: non-ruminant-derived (pig and chicken manure) and ruminant-derived (sheep and cattle manure) substrates. These findings corroborate previous reports that dietary differences fundamentally shape gut microbiome composition, consequently influencing fecal microbial community structures in herbivorous versus omnivorous livestock (Wang et al., 2024).

Notably, Firmicutes dominated composting systems amended with CM and PM, whereas Proteobacteria showed enhanced abundance in SM and CoM. The proliferation of Firmicutes in CM and PM systems can be attributed to their rapid growth kinetics and metabolic versatility in utilizing labile carbon compounds typically abundant in non-ruminant manures (Fu et al., 2025). The predominance of Gram-positive Firmicutes during the thermophilic phase accelerated microbial metabolic activities, resulting in rapid depletion of easily degradable organic matter during early composting stages. This accelerated decomposition cascade consequently promoted elevated emissions of pollutant gases, including CH_4_, N_2_O, and NH_3_, during initial composting phases. During late-stage composting, Actinobacteriota gradually superseded Firmicutes as the dominant phylum in PM, whereas Firmicutes maintained predominance in CM. This differential successional pattern correlated with enhanced N_2_O production in CM, suggesting Firmicutes-mediated nitrogen transformation pathways (Lei et al., 2021).

Conversely, Proteobacteria, specialized in degrading low-molecular-weight organic compounds (Zhu et al., 2024b) and possessing substantial nitrogen-fixing capabilities (Zhang et al., 2022), contributed to reduced NH_3_ and N_2_O emissions in SM and CoM. Furthermore, Actinobacteriota and Bacteroidetes demonstrated specialized metabolic capacities for recalcitrant substrate degradation, including hemicellulose and cellulose decomposition (Feng et al., 2025). Enhanced abundances of these phyla in ruminant manure compostings promoted substrate humification while concurrently reducing H_2_S and CH_4_ emissions, ultimately yielding higher compost maturity indices.

Among the 30 most abundant genera (dominant genera, Figure 3d), CM was dominated by *Thermobifida* (0.90%), *Sporosarcina* (0.50%) and *Bacillus* (0.34%), whereas PM was dominated by *Saccharomonospora* (0.72%), *Thermobifida* (0.53%) and *Bacillus* (0.34%). *Thermobifida* peaked at 0.34% in CM and 0.24% in PM during the thermophilic phase, and its thermostable hydrolases and amylases degrade macromolecules while concurrently enhancing N_2_O emissions (Jia et al., 2023). *Bacillus*, enriched on day 0 in both piles, catalyzes nitrification–denitrification reactions that generate NH_3_ and N_2_O (Wang et al., 2024) and modulates CH_4_ through Fe^2+^-ROS interplay under oxidative stress (Ernst et al., 2022). *Sporosarcina* proliferated in fresh CM, declined during the thermophilic phase and resurged in the cooling-maturation phase. Its activity is stimulated by elevated NH_4_^+^, NO_3_^−^ and pH, intensifying NH_3_ and N_2_O volatilization (Fu et al., 2025). *Saccharomonospora* sustained high abundance in PM across thermophilic conditions within a pH range of 5–11 and promoted H_2_S release (Zhang, Z., et al., 2023).

In SM and CoM profiles, *Thermobifida* and *Saccharomonospora* collectively exhibited the highest total abundance among all genera throughout the composting. *Thermobifida* (0.15%) ultimately dominated the cooling phase in SM (vs. 0.07% in CoM), where its thermostable lignocellulose-depolymerizing enzymes not only drive humification but also exhaust fermentable substrates via cellulolysis. This dual action truncates CH_4_ generation and locks carbon into stabilized humics, collectively yielding a significantly higher germination index (Wen et al., 2021; Zhang, Y., et al., 2025). *Saccharomonospora* peaked at 0.10% on day 28 in SM and persisted at 0.04–0.08% from day 14 to 49 in CoM, an enrichment enabled by phenolic depolymerases and lignocellulolytic activities that thermodynamically stabilize the matrix and accelerate maturation (Ding et al., 2025). *Actinomadura*, scarce in CM and PM, rose to 0.07–0.09% during the maturation of SM and CoM and functions as a critical Actinobacterium that promotes humification, stabilizes substrates, and thereby suppresses N_2_O and odor generation (Xu et al., 2025). Additionally, in SM, *Atopostipes* (0.13% at day 0) has been associated with enhanced lignocellulose degradation and accelerated compost maturation where it is enriched (Yu et al., 2022; Cheng et al., 2025), and is positively correlated with N_2_O reduction potential and inversely associated with CH_4_ emissions during organic waste treatment (Yang et al., 2023). *Flavobacterium* (0.10%) expanded during the thermophilic phase and contributes to lignocellulose deconstruction and biological nitrogen fixation, cutting N_2_O and NH_3_ fluxes (Liu and Zhang, 2023). In CoM, *Psychrobacter* (0.21%) and *Corynebacterium-1* (0.14%) governed the initial microbiome. *Psychrobacter* attenuates NH_3_ and early N_2_O emissions via aerobic respiration and nitrification, whereas *Corynebacterium-1*, an actinobacterium, accelerates organic degradation and maintains aerobic conditions that mitigate CH_4_ and H_2_S emissions while improving C/N and aeration (Li, D., et al., 2022). *Pseudomonas* expanded to 0.09% during the thermophilic phase and operates as a metabolic generalist that couples denitrification to organically heterotrophy, redirecting nitrogen from N_2_O toward N_2_ and reshaping the emission profile. In addition, the broad catabolic armamentarium of *Pseudomonas* oxidizes recalcitrant aromatics and reduced sulphur, indirectly attenuating H_2_S-mediated odor (Liu, Y., et al., 2024).

### 3.4 Variations in bacterial communities associated with gas emissions

Network analysis identified the bacterial genera potentially associated with greenhouse gas and odor emissions across composting systems (Figure 4). Based on these results, the relative abundances of the identified genera (key genera) are further profiled in Figure 5.

**Figure 4:**
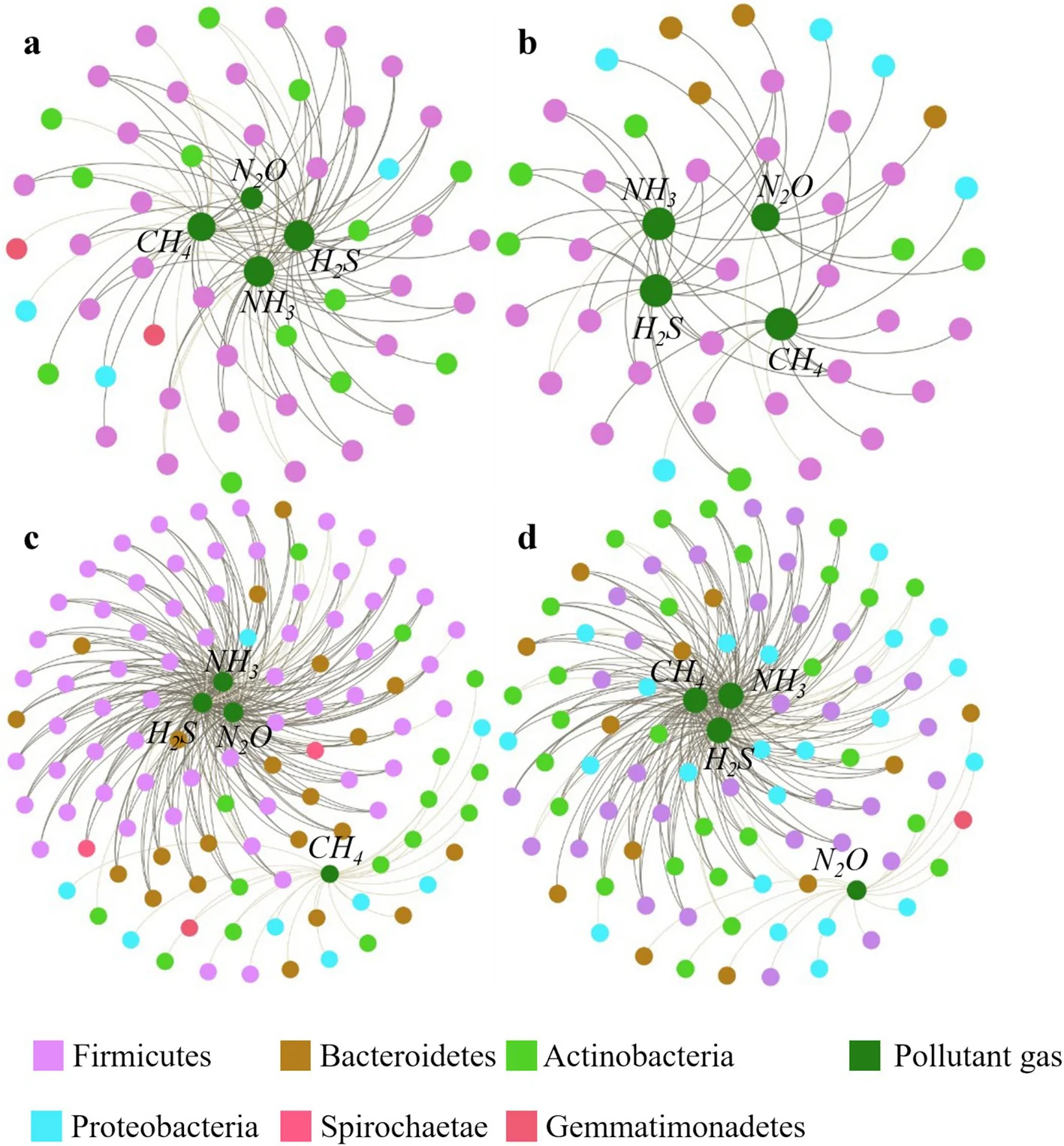
The types of related bacteria genera associated with CH_4_, N_2_O, NH_3_, and H_2_S emission during composting. **(a)** CM, **(b)** PM, **(c)** SM, and **(d)** CoM. CM: chicken manure, PM: pig manure, SM: sheep manure, CoM: cattle manure composting.

**Figure 5:**
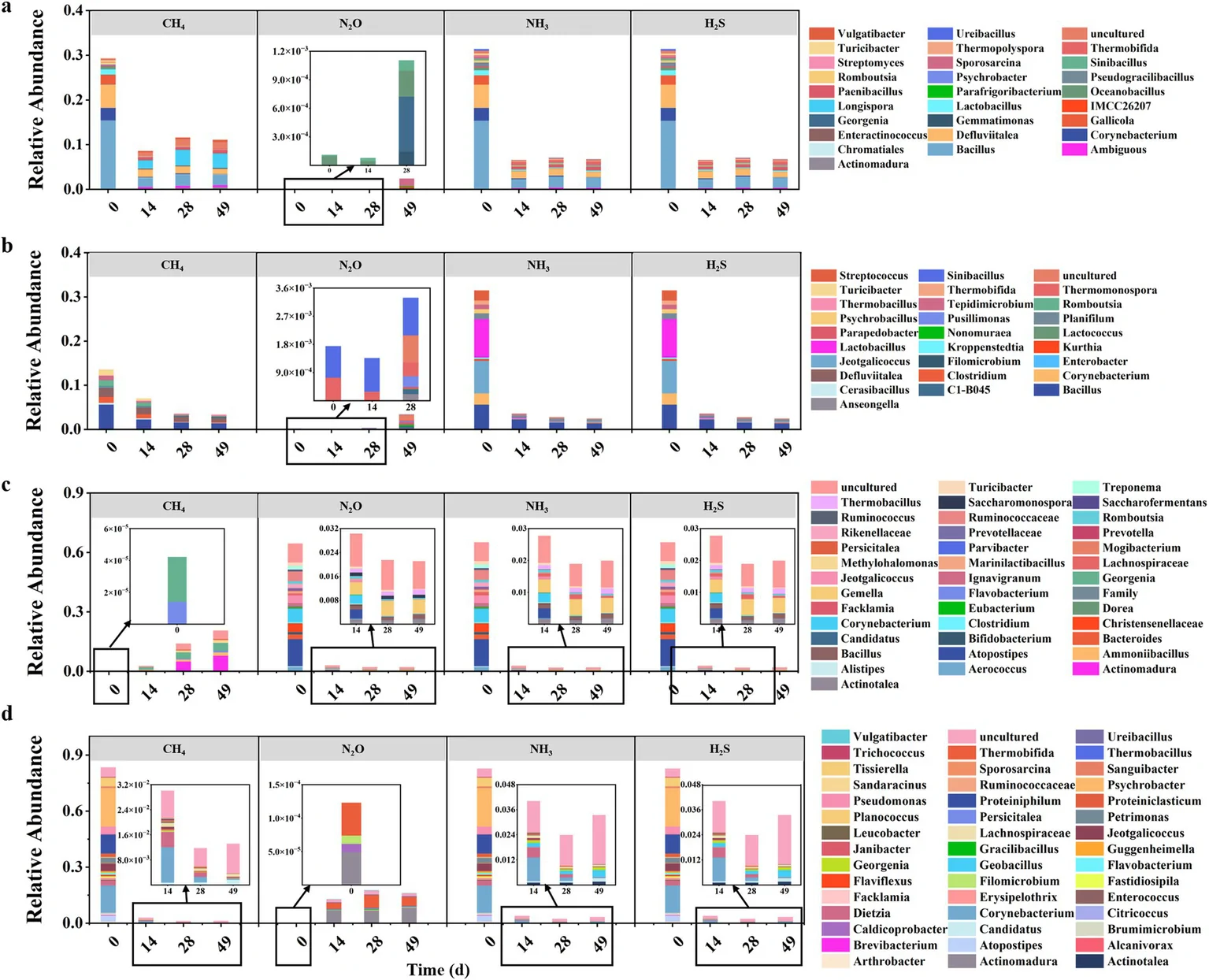
Relative abundance of genera associated with CH_4_, N_2_O, NH_3_, and H_2_S emission during composting. **(a)** CM, **(b)** PM, **(c)** SM, **(d)** CoM. CM: chicken manure, PM: pig manure, SM: sheep manure, CoM: cattle manure composting.

In CM (Figure 5a), *Bacillus* and *Defluviitalea* were the most abundant genera related to CH_4_, NH_3_ and H_2_S emission, while *Sporosarcina* were the most correlated with N_2_O. Moreover, *Bacillus* and *Sporosarcina* also the top abundant genera during CM (Figure 3d). Specifically, the decrease of *Bacillus* and *Defluviitalea* from day 0 to day 49 is presumably a reflection of the rapid decomposition of organic matter in the early composting stage, which might be the major factors contributing to the substantial emission of CH_4_, NH_3_ and H_2_S. Subsequently, the reduction in available degradable substrates leads to a decline in the abundance of methanogenic bacteria (Jiang et al., 2019). Additionally, *Paenibacillus* sustained stable abundances throughout the entire composting period, which points to their potential continuous involvement in proteins degradation and NH_3_ production (Li, H., et al., 2023). *Thermobifida* and *Turicibacter* exhibited a significant increase in abundance on day 49, which underscores their potential function in decomposing organic sulphur compounds and producing H_2_S (Liu, Y., et al., 2024). With respect to N_2_O emission, in addition to *Sporosarcina*, the genera *Georgenia* and *IMCC26207* also reached the peak on day 49, implying their possible participation in nitrogen transformation, including nitrification and denitrification, which in turn affects N_2_O release (Chen et al., 2015). In PM (Figure 5b), *Bacillus* also persisted as the dominant bacterial genera related to CH_4_, NH_3_ and H_2_S emissions, as in CM, and remained the top 30 genera during the composting. For N_2_O emission, *Parapedobacter* exhibited elevated abundance on day 49, implying its potential contribution to N_2_O generation via denitrification (Yang et al., 2020). The relative abundance of microbial genera associated with NH_3_ was similar to that associated with H_2_S. In addition to *Bacillus*, *Lactobacillus* and *Jeotgalicoccus* likewise displayed higher abundance on day 0, which reflected the persistent involvement in organic nitrogen mineralization and subsequent NH_3_ and H_2_S production in the early stage of composting (Jiao et al., 2023).

SM and CoM had significant increase in the number of microbial species associated with gas emissions, and *Actinomadura, Atopostipes* and *Psychrobacter* were also among the top genera during these composting processes (Figures 5c,d). *Actinomadura* may suppress CH_4_ production in SM and N_2_O production in CoM by vigorously consuming readily available carbon sources, thereby depriving methanogens and denitrifiers of their preferred substrates, a mechanism decoupled from lignocellulose degradation (Yang et al., 2026). In addition, *Psychrobacter* and *Corynebacterium* were critical in CoM and were correlated with lower CH_4_ emission, whereas *Atopostipes* and *Corynebacterium* prevailed in SM and were linked to declined N_2_O flux. This difference of *Corynebacterium* with respect to different gases may be attributed to the lower substrate looseness and oxygen content in initial CoM for CH_4_ production, while in the moderately saline SM it might conduct nitrate and nitrite respiration that could fuel N_2_O formation via incomplete denitrification (Yan et al., 2023; Gu et al., 2025). Furthermore, *Atopostipes in* SM and *Psychrobacter* in CoM, as well as *Corynebacterium* in both composting systems, were the key genera associated with the lower NH_3_ and H_2_S emissions. *Psychrobacter*, a psychrotrophic bacterium, may suppress the activity of microorganisms responsible for contaminant gas production through competitive ecological strategies, including nutrient depletion, bacteriocin secretion, and alteration of microenvironmental redox and pH conditions (Liu et al., 2025). *Atopostipes*-driven early-phase remodeling of nitrogen and sulphur metabolic fluxes synchronously suppresses NH_3_ and H_2_S volatilization by channeling ammoniacal-N into biomass and shunting sulfate away from dissimilatory reduction (Liu et al., 2023). Comparing the relative abundance of microbial genera across the different composting, the high abundance of Actinobacteria (including *Thermobifida*, *Actinomadura*) in CoM and SM effectively decomposes complex organic matter, potentially mitigating the rate of CH_4_ production (Xu et al., 2021). Furthermore, the elevated abundance of nitrogen cycle-associated microorganisms (including *Georgenia* and *IMCC26207*) in the late stages of composting promotes the mineralization of organic nitrogen, thereby potentially lowering N_2_O emissions (Hoang et al., 2022). Consequently, gas emissions from CoM and SM were generally lower than those from CM and PM. This observation can also be ascribed to the higher fiber content in CoM and SM, which prolongs the composting period, facilitates a more comprehensive degradation of organic matter, and enhances compost maturity, thus curbing the rapid release of gases (Wang et al., 2023).

### 3.5 Mechanisms linking environmental factors and microorganisms to gas emissions

To explore the potential mechanism underlying gas emissions during composting, we first selected keystone genera associated with CH_4_, N_2_O, NH_3_ and H_2_S emission and examined their pairwise associations with physicochemical factors (pH, EC, GI, temperature, TN, TC, TS, NH_4_^+^, NO_3_^−^) via correlation analysis (Supplementary Figures S2–S5). As a general pattern, NH_4_^+^ and GI emerged as the most frequent correlates with gas-associated taxa, while the specific keystone genera varied distinctly with manure type. *Bacillus* and *Defluviitalea* predominated in CM and PM, whereas *Actinomadura*, *Corynebacterium, Atopostipes* and *Psychrobacter* were more prevalent in SM and CoM. These preliminary correlations, however, only capture bivariate relationships and do not distinguish direct from indirect causal pathways.

To quantitatively disentangle the multi-stage regulatory cascades from environmental drivers to bacterial communities and subsequent gas emissions, we constructed PLS-PM path models for each treatment (Figure 6). This approach allows us to compare the relative strength of direct and indirect effects and to identify divergent regulatory architectures between non-ruminant-derived (CM, PM) and ruminant-derived (SM, CoM) manures.

**Figure 6:**
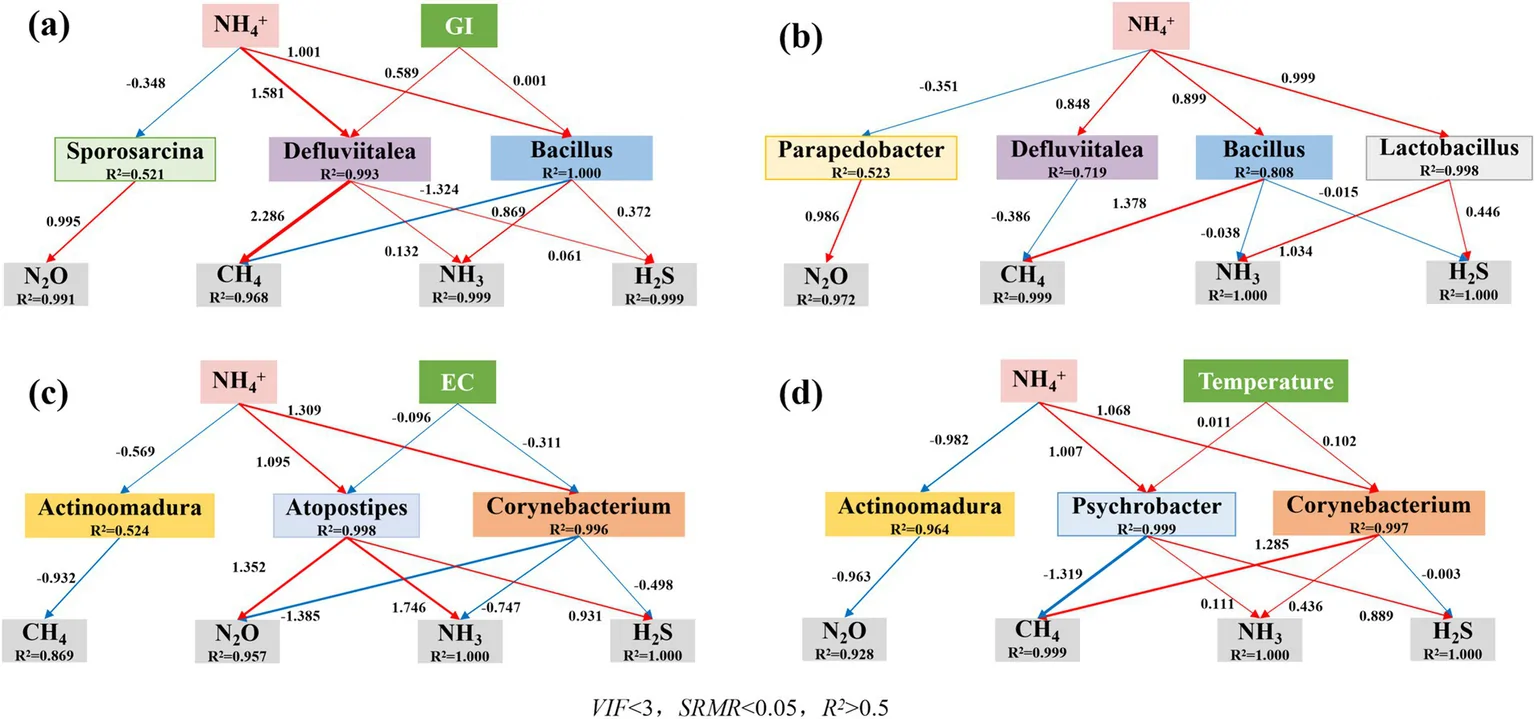
Pathway construction of key related bacteria and environmental factors affecting pollutant gases emission. **(a)** CM, **(b)** PM, **(c)** SM, **(d)** CoM. Red and blue colors represented positive and negative effects. EC: electrical conductivity, GI: germination index, CM: chicken manure, PM: pig manure, SM: sheep manure, CoM: cattle manure composting.

In CM and PM, NH_4_^+^ and GI exerted the strongest direct and indirect effects on gas fluxes, primarily mediated by the keystone genera *Bacillus*, *Defluviitalea* and *Sporosarcina*. The path coefficients for NH_4_^+^ → keystone bacteria → gas emissions were consistently higher than those for other environmental factors, indicating a dominant proximal regulatory role of ammonium availability. PM differed from CM by an additional positive contribution of TC and a slightly negative modulation by pH, which collectively enhanced the relative importance of *Parapedobacter* and *Lactobacillus* in N_2_O, NH_3_ and H_2_S pathways. In contrast, SM and CoM exhibited a distinctly different regulatory topology. Here, temperature and EC, together with NH_4_^+^, formed the primary environmental drivers, and the keystone genera shifted to thermophilic genera (*Actinomadura, Corynebacterium, Atopostipes, Psychrobacter*). Notably, the path coefficients from these bacteria to gas emissions were substantially lower than those observed in CM and PM, consistent with the markedly reduced CH_4_, N_2_O, NH_3_ and H_2_S release rates measured in ruminant manure composts. This suggests that the ruminant-derived matrix may suppress gas production not only through physicochemical conditions but also via a less active functional microbiota.

Of note, the same microbial genera may be associated with multiple gaseous emissions, as they participate in distinct biochemical pathways depending on microenvironmental conditions. For instance, *Corynebacterium* was correlated with reduced CH_4_ emissions in CoM but linked to N_2_O production in SM, reflecting the regulatory effects of substrate properties and oxygen availability on metabolic routing (Yang et al., 2023). Similarly, *Actinomadura* correlated with CH_4_ emission in SM and N_2_O emission in CoM, further illustrating that the metabolic plasticity of microorganisms enables them to assume divergent biogeochemical functions across different ecological niches during composting (Chen et al., 2024; Wang et al., 2023). Additionally, across all four systems, NH_4_^+^ remained the single most influential environmental factor, with consistently high path coefficients (0.72–0.91) toward both bacterial composition and gas emissions, highlighting its universal importance in composting gas regulation. However, the bacterial keystone genera shifted predictably with manure origin: the *Bacillus-Defluviitalea* consortium in non-ruminant manures versus the *Actinomadura-Corynebacterium* consortium in ruminant manures, indicating that host digestive physiology shapes the compost microbiome in ways that substantially affect emission outcomes.

It should be noted that our current findings are based on 16S rRNA gene sequencing, which provides phylogenetic inference but not direct functional confirmation. Therefore, the proposed associations between specific taxa and gas emissions remain correlative rather than causative, and future studies employing metagenomic sequencing, functional gene quantification, or stable isotope probing are warranted to validate these putative microbial roles in gas production pathways.

## 4 Conclusion

This study reveals that manure composting follows two diet-driven regulatory pathways. Non-ruminant manure (chicken, pig), with lower C/N and abundant labile carbon, favor *Bacillus*-dominated communities that accelerate gaseous emissions. Ruminant manures (sheep, cattle), characterized by higher C/N, elevated pH and lignocellulosic content, promote thermophilic actinobacterial consortia (*Actinomadura, Corynebacterium*) associated with enhanced humification and effective nitrogen/sulphur retention (30–93%), resulting in markedly lower emission potentials. Across all composting systems, NH_4_^+^ serves as the most consistent proximal driver of gas fluxes, yet its regulatory outcomes are strongly modulated by feedstock origin. This implies that uniform mitigation strategies are unlikely to be universally effective. Instead, tailored approaches, such as bulking agent optimization for non-ruminant manures and temperature-moisture management for ruminant manures, are required. Our findings provide a theoretical basis for feedstock-specific emission reduction in composting operations. Future functional validation (e.g., metagenomics) is needed to confirm the causative roles of keystone taxa and to enable targeted microbiome interventions for cleaner composting.

## Data Availability

The datasets presented in this study can be found in online repositories. The names of the repository/repositories and accession number(s) can be found in the article/Supplementary material.
