# Supplementary material for: Ruminant versus non-ruminant manure composting: carbon-to-nitrogen ratio and associated physicochemical traits impact divergent pollutant emissions
Source: Front Microbiol. 2026 Sep 25;17:1890442. doi: 10.3389/fmicb.2026.1890442 (PMC13649995; doi:10.3389/fmicb.2026.1890442)
Supplement: Supplementary file 1 [file Data_Sheet_1_(6).docx]

**Fig. S1** Changes of (a) moisture and (b) CO_2_ content during composting. CM: chicken manure, PM: pig manure, SM: sheep manure, CoM: cattle manure composting.

**Fig. S2** The correlation between the key bacteria and environmental factors in chicken manure composting (CM). (a) CH_4_, (b) N_2_O (c) NH_3_ and (d) H_2_S emissions. Asterisks indicate significant (*, *P* < 0.05), highly significant (**, *P* < 0.01), and very highly significant (***, *P* < 0.001) relationships. EC: electrical conductivity, GI: germination index, T: temperature, TN: total nitrogen, TC: total carbon, TS: total sulphur.

**Fig. S3** The correlation between the key bacteria and environmental factors in pig manure composting (PM). (a) CH_4_, (b) N_2_O (c) NH_3_ and (d) H_2_S emissions. Asterisks indicate significant (*, *P* < 0.05), highly significant (**, *P* < 0.01), and very highly significant (***, *P* < 0.001) relationships. EC: electrical conductivity, GI: germination index, T: temperature, TN: total nitrogen, TC: total carbon, TS: total sulphur.

**Fig. S4** The correlation between the key bacteria and environmental factors in sheep manure composting (SM). (a) CH_4_, (b) N_2_O (c) NH_3_ and (d) H_2_S emissions. Asterisks indicate significant (*, *P* < 0.05), highly significant (**, *P* < 0.01), and very highly significant (***, *P* < 0.001) relationships. EC: electrical conductivity, GI: germination index, T: temperature, TN: total nitrogen, TC: total carbon, TS: total sulphur.

**Fig. S5** The correlation between the key bacteria and environmental factors in cattle manure composting (CoM). (a) CH_4_, (b) N_2_O (c) NH_3_ and (d) H_2_S emissions. Asterisks indicate significant (*, *P* < 0.05), highly significant (**, *P* < 0.01), and very highly significant (***, *P* < 0.001) relationships. EC: electrical conductivity, GI: germination index, T: temperature, TN: total nitrogen, TC: total carbon, TS: total sulphur.
